# Subjective consistency increases trust

**DOI:** 10.1038/s41598-023-32034-4

**Published:** 2023-04-06

**Authors:** Andrzej Nowak, Mikolaj Biesaga, Karolina Ziembowicz, Tomasz Baran, Piotr Winkielman

**Affiliations:** 1 Robert Zajonc Institute for Social Studies, University of Warsaw, Warsaw, Poland; 2 Institute of Psychology, The Maria Grzegorzewska University, Warsaw, Poland; 3 Faculty of Psychology, University of Warsaw, Warsaw, Poland; 4 University of California, San Diego, La Jolla, CA USA; 5 Faculty of Psychology, SWPS University of Social Science and Humanities, Warsaw, Poland; 6 Department of Psychology, Florida Atlantic University, Boca Raton, USA

**Keywords:** Psychology, Human behaviour

## Abstract

Trust is foundational for social relations. Current psychological models focus on specific evaluative and descriptive content underlying initial impressions of trustworthiness. Two experiments investigated whether trust also depends on subjective consistency—a sense of fit between elements. Experiment 1 examined how consistency of simple verbal characterizations influences trust judgments. Experiment 2 examined how incidental visual consistency impacts trust judgments and economic decisions reflecting trust. Both experiments show that subjective consistency positively and uniquely predicts trust judgments and economic behavior. Critically, subjective consistency is a unique predictor of trust that is irreducible to the content of individual elements, either on the dimension of trust or the dimension of valence. These results show that trust impressions are not a simple sum of the contributing parts, but reflect a “gestalt”. The results fit current frameworks emphasizing the role of predictive coding and coherence in social cognition.

## Introduction

Who would you initially trust more? A shy salesperson or an outgoing salesperson? A businessperson in the office or on a beach? A slim cook or a plus-size cook? In the current work, we investigate the psychology of initial impressions of trustworthiness, and propose that they depend not only on the content of the stimulus, but also on a sense of fit—subjective consistency—between different components of the stimulus or the stimulus and its context. Before explaining our particular theoretical and empirical approach, we position our work within the larger literature on trust.

The topic of trust is central for many disciplines in the social sciences and the subject of a vast literature and lively debates. This is because trust underpins our society. Politically, it supports democracy and citizen's well-being^1^. Economically, trust enables transactions and allows common resources^2,3^. Relations at the interpersonal level require that individuals believe their partners will keep their promises, honor agreements, and conform to norms. In short, people need to trust others. People also want to be trusted. The reputation of being trustworthy contributes to the social capital of an individual, as it enhances chances that others will cooperate with them, follow them, and give access to critical resources^4,5^. Moving beyond the human–human side of trust, an emerging literature highlights the importance of trust in relation to non-human targets, such as animals and technology, including cars, robots and AI^6–8^.

Reflecting this broad relevance, definitions and models of trust highlight different dimensions and determinants. The sociological literature focuses on trust in the system, or the beliefs that societal structures and institutions support individual activities^9^. Within the broad literature on interpersonal relations, often coming from the field of organizational behavior, the common dimensions and determinants of trust are competence (ability), benevolence (warmth), and integrity, in the sense of honoring agreements^10^. Some authors additionally highlight the dimension of predictability, in the sense of a perceiver's ability to forecast a target's behavior based on a reliable pattern^8^. These dimensions can be assessed at the level of social systems, organizations, and interpersonal relations. However, within an individual, they underpin trust as a "psychological state comprising the intention to accept vulnerability based upon positive expectations of the intentions or behavior of another"^11^. Notably, some authors highlight that such a rich, intentional definition of trust is too restrictive for capturing initial trust impressions, and trust relations with non-human entities, especially technology^8^. As such, we will treat trust as a broad psychological attitude, as described shortly.

The existing trust literature also helpfully distinguishes *judgments of trustworthiness* (assessments of potential targets) from *trust decisions*, which additionally incorporate features of the particular situation, knowledge about the system, and individual differences in general willingness to trust, some of which may have biological bases^12^. Importantly, many researchers in this literature emphasize the importance of considering both cognitive and affective elements of trust^13,14^. They also recognize a continuum ranging from *distrust*, via a more neutral *untrust*, to genuine *trust*, with some attitudes and behaviors reflecting only minimalistic, rudimentary forms of trust^15^. Within this broad tradition, our research represents efforts trying to understand the psychology of trust when social perceivers are trying to form initial impressions.

Our particular focus is grounded in the tradition of social psychology, which focuses on first impressions of anonymous strangers, which is an initial step in the formation of attitudes^16^. Specifically, we are interested in general personal impressions of trustworthiness—a concept describing the perceived characteristics of the trustee. Originating in social psychological research on the role of credibility, the concept of "trustworthiness" assumes that social perceivers form broad cognitive and affective expectations about the qualities of potential interactants^17–19^. These broad impressions, which like attitudes have cognitive, affective, and behavioral components, can be expressed in trustworthiness judgments. These impressions can be measured by trust ratings of targets, and specific trust behaviors, for example decisions made in economic games. In our work, we assume that these judgments and behaviors can derive from minimal sources of information about the described target individual. This assumption is grounded in the rich psychological literature on peripheral cues in persuasion^16^ and on the role of thin slices and simple features in forming impressions^20,21^.

### Central and peripheral cues of trust

So, how do people evaluate the trustworthiness of other individuals? With acquaintances, the history of personal interactions matters most^22^. The just discussed dimensions of trust—competence, benevolence, integrity, and predictability—can be learned in the course of repeated interpersonal interactions^10^. With strangers, perceivers tend to start with "default" trust [^23^, but see^24^], which is qualified by additional cues. Some come from social knowledge, including reputation and stereotypes. For example, some occupations, social positions and roles (e.g. nurses, doctors, and teachers) are trusted while others (e.g. politicians, truck-drivers, and car salespersons) are distrusted^25^. In addition, many trait terms contain cues about trust and the correlated dimension of warmth: a *kind nurse* is trusted more than an *aggressive nurse*^26^.

Crucial for our approach here, people also evaluate trust using peripheral features. For example, research on "thin-slice" interactions shows that structural facial features, such as roundness, and emotional features, such as a smile, increase trust^27–29^. In contrast, some structural face features such as face-width (relative to facial height) decrease trust^30^. This research also shows that the trust dimension is highly related to valence^21^. Again, as information about the target increases, perceivers’ trust judgments reflect a combination of peripheral cues with personal experience and broader social knowledge^22^.

### Current work

The current work explores how perceivers form trust judgments and behaviors towards minimally described targets from a combination of simple cues. Critically, we argue that when judging trust, perceivers attend not only to the content of discrete trust cues, but also to the internal consistency of that information. Consequently, when individual elements fit together, the target is trusted more than when the fit is low. Our proposal has several key implications. First, it implies that the final trust judgment is more than a sum of its parts and cannot be fully predicted from the evaluative and descriptive characteristics of individual elements. As such, it challenges models that primarily link trust to specific affective or descriptive content. Second, and related, we propose that perceivers rely on their subjective sense of consistency, which again is not reducible to the characteristics of individual contributing elements but instead reflects an assessment of a general fit. To explain our reasoning, we first elaborate on the notion of consistency, and then the notion of subjective experience.

### Consistency

Consistency is a key term in social psychology. It is famously explored in theories of dissonance and impression formation [for review^31^]. More recently, it is applied in research on social influence^32^ and in work linking social impression formation to predictive coding frameworks^33,34^. We briefly review the key concepts next. Note that social psychologists use the term *consistency* in its logical sense, described below, rather than in a more practical sense, as implying reliability or constancy in behavior.

Asch^35^ noted that personality impressions are not simply additive but a function of consistency between multiple traits. Festinger^36^ proposed that two cognitive elements are inconsistent if one implies the opposite of the other. Critically, he distinguished between *objective inconsistency* (simultaneously believing p and not-p) and *subjective inconsistency* (a sense of dissonance). Closely related to this notion is *coherence,* defined as maximal satisfaction of multiple constraints, or an overall fit between elements^37^. Though used differently in philosophy, we use *consistency* similarly to *coherence*. For example, accepting that someone is "beautiful" may psychologically imply that this person is "kind" (positive constraint) but rejects "hateful" as a characteristic (negative constraint). One distinction often drawn in this literature is between *evaluative* and *descriptive* consistency. Characterizing someone as a "beautiful hater" is evaluatively inconsistent, because the elements are opposite in valence. Characterizing someone as a "tall dwarf" or a "slim cook" is descriptively inconsistent, because the elements seem contradictory or incongruent. Both evaluative and descriptive consistency can drive social judgments. For example, evaluative consistency of the target with the context strengthens moral character impressions^38^. Regarding descriptive consistency, banners on a website generate more trust when they are topically congruent versus incongruent^39^.

Consistency and coherence can work as cues on multiple levels. Inconsistency disturbs the maintenance of life meaning—a sense of trust that life makes sense^40,41^. In law, consistency within a witness' testimony enhances credibility^42^. In science, credibility comes from consistency with evidence and explanatory coherence. In persuasion, inconsistency reduces the credibility of the message and the source. On a low level, inconsistency between nonverbal channels is a cue to lying^43^. Such rudimentary inconsistency can also be a source of negative affect, as it leads to greater processing effort^44–46^.

More fundamentally, cognitive science suggests that consistency evaluation is a basic feature of mental life. For example, predictive coding theories propose that pre-existing knowledge is combined with the current stimulus environment to build a model of the world and derive predictions about what will happen next^33,34,47^. Inconsistent information represents a "prediction error", and suggests that the existing model of the world needs to be revised. As such, inconsistency signals unpredictability. It can therefore reduce trust, because a precondition of trust is being able to anticipate the target’s behavior. Importantly, these newer theoretical approaches to consistency highlight that predictability interacts with information value^34,48^. For example, if a negative element (e.g., *thief*) is paired with a positive element (e.g. *honest*), the negative "surprise" of inconsistency is reduced by a positive reaction to the overall outcome (e.g., a thief returns a stolen wallet). In short, perceivers' final trust judgments should reflect information about the target’s descriptive and evaluative features, as well as consistency of this information. One example for this interplay between the specific content of features and their internal consistency is research on trust judgments from faces varying in emotional expression. As mentioned, participants rely on smiling features, generally trusting happier faces more. However, increasing the amount of smiling features does not always enhance trust, if adding those smiling cues leads to an internal conflict with other facial features (e.g., anger features), creating internal inconsistency and greater processing effort^49,50^.

### Objective consistency and the feeling of subjective consistency

Researchers distinguish between "objective" and "subjective" inconsistency. Objective inconsistency is a mismatch between the evaluative and descriptive meaning of individual elements constituting a description of a target. In our case, the objective inconsistency could be on valence (one element is positive and the other is negative) or trust (one element implies high trust and the other implies low trust) or both. For example, "*corrupt nurse*" is objectively inconsistent on both valence and trust because the element *corrupt* connotes low trust and is negative whereas *nurse* connotes high trust and is positive. However, as Asch^35^ and Festinger^36^ emphasized, inconsistency also has a psychological meaning. For example, a "slim cook" is evaluatively consistent (both elements are independently positive). It is also descriptively consistent on the dimension of trust (both "slim" and "cook" are neutral in terms of reputation). However, the combination may be psychologically, or subjectively inconsistent, since people *expect* cooks to be fat. Indeed, what matters in cognitive conflict is subjective inconsistency, rather than objective contradiction^51^. Current research explores this issue in the domain of trust judgments.

Finally, since James^52^ and Festinger^36^ researchers have emphasized that people have a "sense of inconsistency"—a subjective experience that communicates the overall quality of mental processing. This subjective experience can efficiently represent rich relational information^53^. Indeed, people rely on "structural experiences" such as a sense of coherence, fluency, integrity, or rightness to make a variety of judgments^49,54,55^. Interestingly, this sense of inconsistency can arise incidentally, induced by background stimulus features, yet still influence target judgments^56^. Accordingly, in the current research we explore how trust judgments depend on this general sense of consistency, even when induced by incidental features.

### Current studies

The preceding discussion suggests that many different theoretical considerations converge on the following prediction: Subjective consistency should serve as an input into trust judgments, and trust should increase with greater subjective consistency. The current studies examined this hypothesis using a simple paradigm where basic components of a stimulus varied in consistency.

In Experiment 1, we asked participants to judge their trust in targets described by a pair of words—an adjective and a noun that varied on consistency. The goal here was to manipulate consistency *within* the same person target (described by an adjective and a noun). We did it by pairing the target person (noun) with descriptions (adjectives) that either fit the person (noun) or not (*Supplementary Table* 1).

In Experiment 2, we asked participants to play an economic Trust Game with, or judge trust of, visual targets—pictures of people presented on backgrounds varying in incidental consistency. This was important for three reasons. First, we wanted to assess the role of consistency between the target and its context (rather than within the target itself). Second, we wanted to examine if sources of inconsistency can also be visual. Third, we wanted to move beyond trust judgments to a trust behavior that involves a decision to accept vulnerability to the target person, as implied in the trust definition by Rousseau et al.^11^ and traditionally studied in economic trust games^23^.

In each experiment, we also independently measured and examined the role of subjective inconsistency and its relation to objective measures of inconsistency in stimulus features. This independent measurement was designed to avoid a possible bias from a bleed-over of consistency judgments into judgments of trust. Information about how our sample size was determined for each study, a discussion of statistical power, and data exclusions are presented in *Supplementary Materials*.

## Experiment 1

This experiment investigated how internal consistency of a target's verbal description influences trust judgments. This experiment consisted of three separate sub-studies (each study having an independent participants sample) aimed at separately assessing (i) trust and valence of individual components of a target description (Study 1A), (ii) subjective consistency of the compound target description (Study 1B) and (iii) trust judgments for the target description (Study 1C). Our final analyses then combine the three sub-studies (samples) to assess the interrelationship between the key variables.

## Methods

### Design and participants

Study 1A had 50 participants (10 males, MAge = 29.9, SDAge = 8.32). Study 1B had 122 participants (61 males, MAge = 29.5, SDAge = 5.93). Study 1C had 106 participants (53 males, MAge = 28.8, SDAge = 5.54). Each person participated only in one of the studies. For Study 1A, participants came from a SWPS University in Poland. In Studies 1B and 1C responses came from the Polish Nationwide Online Public Opinion Poll Panel *Ariadna*. All participants were Polish and were tested in their native Polish language. Participants responded to our research questions that were embedded in an unrelated larger public opinion survey. The administrators of *Ariadna Panel* employ an internal system of checks to ensure that participants understand the materials and provide quality data.

The procedure was approved by the ethics committee of the Robert Zajonc Institute for Social Studies at the University of Warsaw. Before the survey, all participants were informed about the procedure and told that they could withdraw at any point. All participants gave informed consent and were rewarded with credits (in Study 1A—they received academic credits, in Studies 1B and 1C Ariadna Panel rewarded them with credits that could be exchanged for small gifts). All methods and procedures were performed in accordance with the relevant guidelines and regulations.

### Procedure

In Study 1A, participants saw a list of 91 different words (45 adjectives and 46 nouns) selected in the pilot studies described in *Supplementary Materials*. These words were used to construct the pairs in Studies 1B and 1C. In Study 1 A, each word was presented separately and was rated on trust and valence using 5-point Likert scales, ranging from − 2 to + 2. The specific questions were "How much would you trust a person characterized by this word?" and "How negative/positive is this word for you?".

In Studies 1B and 1C, participants were presented with 64 adjective-noun pairs. In Study 1B, participants assessed each pair’s subjective consistency by answering the question: "How well do these words fit together?" In Study 1C, participants indicated trust towards the described person by answering the question "How much would you trust a person characterized by these words?" In both studies, participants made their judgments on 5-point Likert scale. As mentioned, the reason to assess all these measures separately was to avoid the carry-over from the ratings of individual stimulus components, and ratings of consistency, to our main measure of trust, which could raise issues of demand characteristics or halo effects. The item content and inter-item correlations are provided in *Supplementary Materials*.

### Analysis strategy

We analyzed the data on the level of individual words and their combinations (pairs). That is, for each single word we had its valence rating and trust rating (Study 1A). For each pair we also had its subjective consistency rating (Study 1B) and its trust rating (Study 1C). With this information, we mean-centered each variable at the level of a person. Hence, we transformed the raw variables from Study 1A to the judgement of valence and trust at the level of words (with the mean 0 for every person and the standard deviation 1). The same transformation was performed in Study 1B and Study 1C. In both studies, we transformed the raw data to the Subjective Consistency (with the mean 0 for every single person and the standard deviation 1) and Judgement of Trust (with the mean 0 and the standard deviation 1) at the level of pairs. With these mean-centered values, we computed the average ratings of valence and trust at the level of words (from Study 1A), and the average measures of consistency (from Study 1B) and trust (from Study 1C) at the level of pairs. Therefore, we had each pair described by the average measure of consistency and trust. Each single word was described by the average measures of valence and trust.

From the above-mentioned measures at the level of words, we computed two indices to assess objective consistency at the level of pairs as a combination of ratings at the level of single words. The Consistency of Trust was a dummy variable that took the value 0 when the average rating of noun's trust had an opposite sign than the average rating of adjective’s trust, otherwise, the value was 1 (pair was consistent in terms of trust ratings at the level of words). The Consistency of Valence was a dummy variable that took the value 0 when the average rating of noun's valence had an opposite sign than the average rating of adjective’s valence, otherwise, the value was 1 (pair was coherent in terms of valence ratings at the level of words). The Mean of Trust at the level of pair was the average of the noun’s trust and the adjective’s trust. The Mean of Valence at the level of pair was the average of the noun’s valence and the adjective’s valence.

### Results

We present the results in two parts. The first part presents simple relationships between judgments of trust and individual predictors—subjective consistency of the pair, mean valence of each word of the pair, and mean trust of each word of the pair. These analyses give a general overview of the results. The second part presents more detailed analyses aimed at assessing the relative importance of each component and their interrelationship.

### Simple predictors of trust judgments

**Figure 1:**
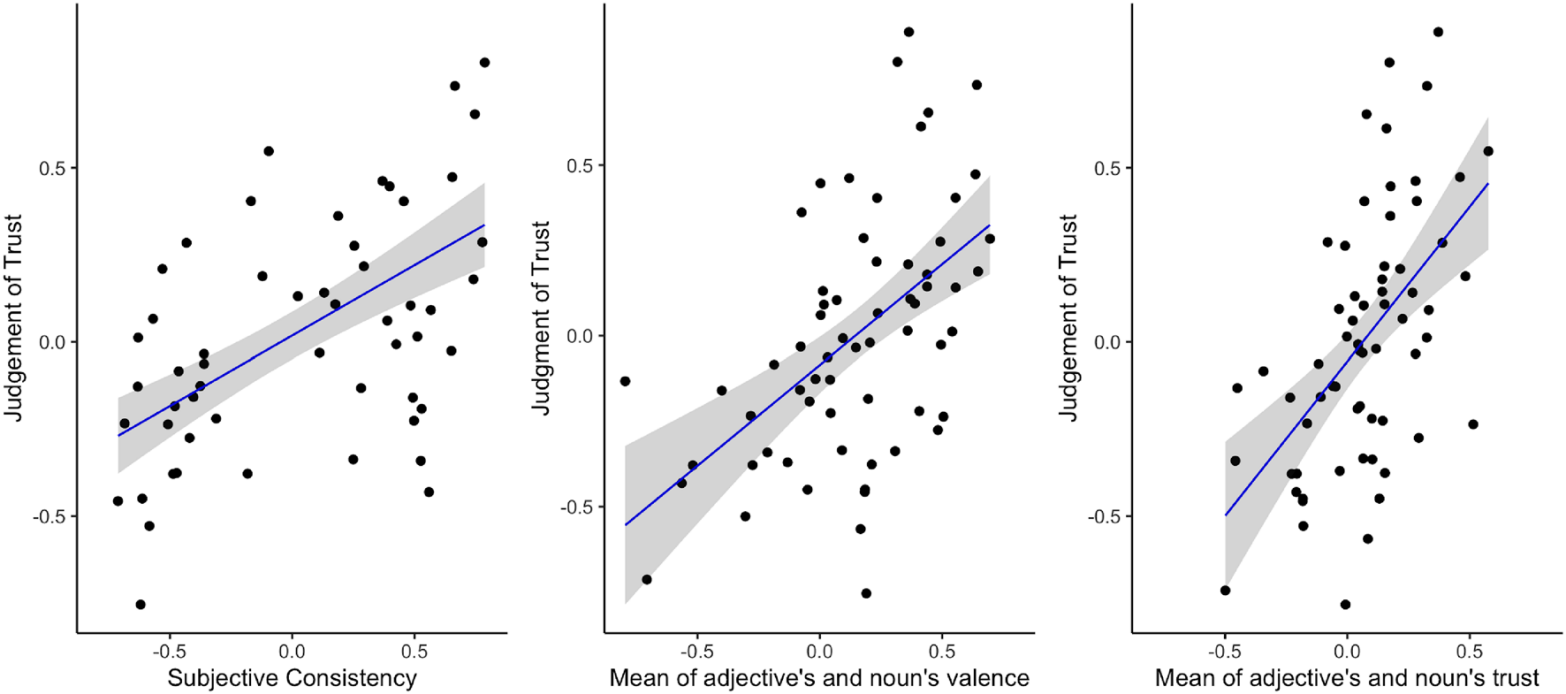
Left panel: The trend line with 95% confidence interval for the relationship between the Judgement of Trust and the Subjective Consistency, R^2^ = 39.83. Central panel: The trend line with 95% confidence interval for the relationship between the Judgment of Trust and the Mean of adjective’s and noun’s valence, R^2^ = 28.47. Right panel: The trend line with 95% confidence interval for the relationship between the Judgement of Trust and the Mean of adjective’s and noun’s trust, R^2^ = 28.33.

For testing the simple relationship between trust and its predictors, we used a weighted least square linear regression model (weights were inversely proportional to the variance at the level of each pair for trust). The dependent variable was Judgement of Trust at the level of pairs, and each independent variable was entered separately—Subjective Consistency, Mean item valence (mean of adjective and noun), and Mean item trust (mean of adjective and noun). When describing statistics below, numbers in square brackets [] show 95% confidence intervals (CI). Figure 1 shows the results.

As shown in the left panel, the model revealed a significant effect of Subjective Consistency on Judgement of Trust. With a 1 point increase of Subjective Consistency there was a 0.40, [CI 0.28, 0.527] increase of Judgement of Trust, *F*(1,62) = 42.7, *p* < 0.001. Subjective Consistency explained about 40% of the variance in Judgment of Trust. As shown in the middle panel, the model also yielded a significant effect of Mean adjective’s and noun’s valence on Judgement of Trust. With a 1 point increase of the Mean of adjective’s and noun’s valence there was a 0.59, [CI 0.359, 0.821] increase of Judgement of Trust, *F*(1, 62) = 26.07, *p* < 0.001. Mean valence of pairs' components explained about 28% of variance in trust judgments. As shown in the right panel, the model revealed a significant effect of the Mean of adjective’s and noun’s trust on Judgement of Trust. With a 1 point increase of the Mean of adjective’s and noun’s trust there was a 0.89 [CI 0.539, 1.236] increase of the Judgement of Trust, *F*(1, 62) = 25.9, *p* < 0.001. Mean trust of pairs' components explained 28% of variance in trust judgments.

### Relations between subjective consistency and other predictors of trust judgments

The more interesting questions relate to whether subjective consistency plays a unique role in trust judgments. That is, does subjective consistency contribute to trust beyond the ratings of individual stimulus components (on trust, or on valence)? Is subjective consistency accounted for by the component combination? Furthermore, is subjective consistency a mediator of the relationship between judgments of trust and objective measures of consistency—defined as a match (lack of conflict) between the independently rated trust and valence of each separate component (explained shortly). To address these questions, we performed two mediation analyses.

First, we tested a mediation model in which Subjective Consistency mediated effects of Noun’s Trust, Adjective’s Trust and Objective Consistency of Trust on Judgment of Trust. We defined the Objective Consistency of Trust as a lack of conflict between the independently rated trust of each stimulus component—a dummy variable that took the value 0 when the rating of Noun's Trust had an opposite sign than the rating of Adjective’s Trust, otherwise, the value was 1. Here (and in later studies) the 95% confidence intervals of indirect effects were estimated with 1000 bootstrap sample.

As shown in Table 1, Judgement of Trust was correlated with Subjective Consistency, Noun’s Trust and Adjective’s Trust, but not with Objective Consistency of Trust. Interestingly, Objective Consistency of Trust was not correlated with Subjective Consistency.

**Table 1:** Intercorrelations of the variables in the first mediation model.

	Judgement of trust	Subjective consistency	Noun’s trust	adjective’s trust
Subjective consistency	0.622***			
Noun’s trust	0.369**	− 0.004		
Adjective’s trust	0.373**	0.199	− 0.108	
Objective consistency of components on trust	0.144	0.061	0.02	− 0.032

*p < 0.05; ** < 0.01; *** < 0.001.

**Figure 2:**
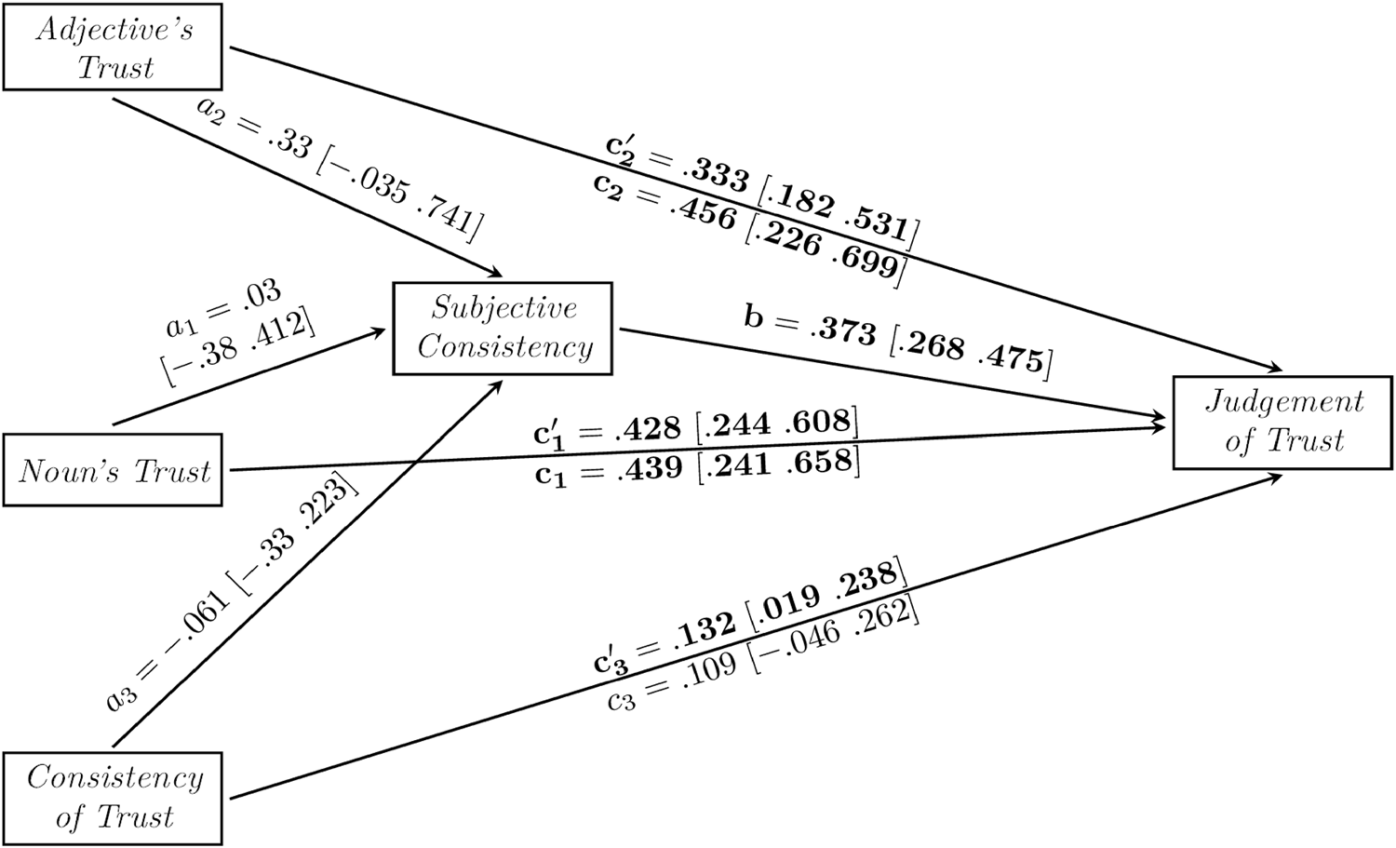
The mediation model of the relationship between the Objective Consistency of Trust (with the inconsistent pair being the reference level in dummy coding), Noun’s Trust, Adjective’s Trust, Subjective Consistency and Judgement of Trust. The fitted model explains 64.6% of variance in Judgement of Trust. Numbers in square brackets show 95% confidence intervals.

As shown in Fig. 2, in the total effect model (without a mediator), both effects of Noun’s Trust (c1 = 0.439, [CI 0.241, 0.658]) and Adjective’s Trust (c2 = 0.456, [CI 0.226, 0.699]) on Judgement of Trust were significant. Only the effect of Objective Consistency of Trust (c3 = 0.109, [CI − 0.046, 0.262]) was non-significant. All three predictors in the total effect model explained 33.1% of variance in Judgement of Trust.

In the next step, we tested whether Noun’s Trust, Adjective’s Trust and Objective Consistency of Trust predicted Subjective Consistency. All three effects, Noun’s Trust (a1 = 0.03, [CI − 0.38. 0.412]), Adjective’s Trust (a2 = 0.33, [CI − 0.035, 0.741]), and Objective Consistency of Trust (a3 = − 0.061, [CI − 0.33, 0.223]) were non-significant. They explained only 4.3% of variance in Subjective Consistency.

After the mediator, Subjective Consistency, was included in the model, the direct effects of Noun’s Trust (c’1 = 0.428, [CI 0.244, 0.608]), Adjective’s Trust (c’2 = 0.333 [CI 0.182, 0.531]), and Objective Consistency of Trust (c’3 = 0.132, [CI 0.019, 0.238]) were significant. Notice that the effect of Subjective Consistency was also a positive and significant predictor of Judgement of Trust (b = 0.373, [CI 0.268, 0.475]) even when the other variables were included in the model. None of the indirect effects of Noun’s Trust (a1b = 0.011, [CI − 0.145, 0.168]), Adjective’s Trust (a2b = 0.123, [CI − 0.013, 0.289]) nor Objective Consistency of Trust (a3b = − 0.023, [CI − 0.126, 0.084]) were significant. The mediation model explained 64.6% of variance in Judgement of Trust.

Beside the expected finding that overall trust judgments are predicted by trust in individual trust components, the key take-away points from this analysis is that subjective consistency is also a unique predictor of judgment of trust and is different than objective consistency.

Second, we computed a mediation model in which Subjective Consistency mediated effects of Noun’s Trust, Adjective’s Trust and Objective Consistency of Valence (a dummy variable that took the value 0 when the average rating of noun’s valence had an opposite sign than the average rating of adjective’s valence, otherwise, the value was 1) on Judgement of Trust. Table 2 shows that Judgment of Trust was correlated with all variables in the model: Subjective Consistency, Noun’s Trust, Adjective’s Trust, and Objective Consistency of Valence. Moreover, Objective Consistency of Valence was also correlated with Subjective Consistency and Adjective’s Trust. Therefore, it is likely that one of them mediates its effect on the Judgement of Trust.

**Table 2:** Intercorrelations of the variables in the second mediation model.

	Judgement of trust	Subjective consistency	Noun’s trust	Adjective’s trust
Subjective consistency	0.622***			
Noun’s trust	0.369**	− 0.004		
Adjective’s trust	0.373**	0.199	− 0.108	
Objective consistency of valence	0.33**	0.37**	0.06	0.33**

*p < 0.05; ** < 0.01; *** < 0.001.

**Figure 3:**
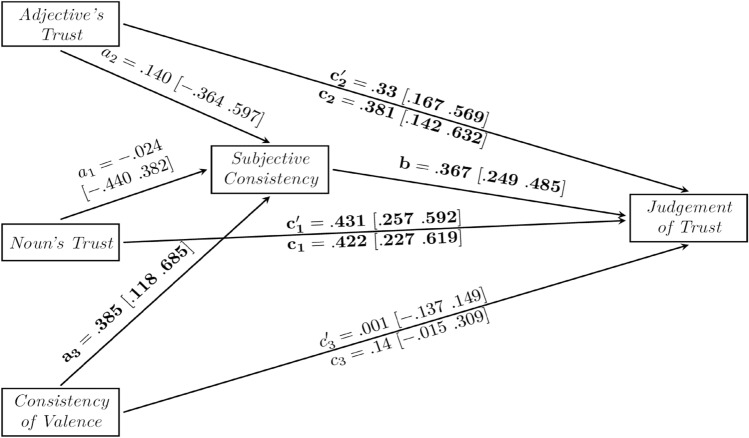
The mediation model of the relationship between Objective Consistency of Valence (with the inconsistent pair being the reference level in dummy coding), Noun’s Trust, Adjective’s Trust, Subjective Consistency and Judgment of Trust. The fitted model explains 61.3% of variance in judgments of trust. Numbers in square brackets show 95% confidence intervals.

As shown in Fig. 3, in the total effects model (without mediator), both effects of Noun’s Trust (c1 = 0.422, [CI 0.227, 0.619]) and Adjective’s Trust (c2 = 0.381, [CI 0.142, 0.632]) on Judgement of Trust were significant. Only the effect of Objective Consistency of Valence was non-significant (c3 = 0.14, [CI − 0.015, 0.309]). All three predictors in the null model explained 34.1% of variance in Judgment of Trust.

In the next step, we tested whether Noun’s Trust, Adjective’s Trust and the Objective Consistency of Valence predicted Subjective Consistency. Although the effects of Noun’s Trust (a1 = − 0.024, 95% CI [− 0.440, 0.382]) and Adjective’s Trust (a2 = 0.140, 95% CI [− 0.364, 0.597]) on Subjective Consistency were non-significant, the effect of the Objective Consistency of Valence was significant (a3 = 0.385 [0.118 0.685]). All three predictors explained 14.2% of variance in Subjective Consistency.

After the mediator, Subjective Consistency, was introduced to the model, it yielded a positive effect (b = 0.367, [CI 0.249, 0.485]) on Judgement of Trust. The direct effects of both Noun’s Trust (c’1 = 0.422, [CI 0.227, 0.619]) and Adjective’s Trust (c’2 = 0.381, [CI 0.142, 0.632]) on Judgement of Trust were significant while the direct effect of Objective Consistency of Valence (c’3 = 0.001, [CI − 0.137, 0.149]) was not significant. However, unlike non-significant indirect effects of Noun’s Trust (a1b = 0.009 95% CI [− 0.156 0.159]) and Adjective’s Trust (a2b = 0.051 95% CI [− 0.141 0.223]), the indirect effect of the Objective Consistency of Valence (a3b = 0.141 95% CI [0.037 0.282]) on the Judgement of Trust was significant. The mediation model explained 61.3% of the Judgment of Trust variance.

Therefore, the impact of Objective Consistency of Valence, unlike the Objective Consistency of Trust in the first model, is fully mediated by the Subjective Consistency. It means that Objective Consistency of Valence (as the combination of positive–negative valence at the level of words) is just a part of the Subjective Consistency, which in turn explains most of the Judgement of Trust. The effect of trust at the level of separate words improve the model, however their effects are direct on Judgment of Trust, and are not mediated by Subjective Consistency.

## Discussion

Experiment 1 showed that on the level of simple correlations, trust judgments are well predicted by subjective consistency. This is important because knowing the subjective consistency of the target description predicts as well as knowing the mean valence and mean trust for individual components—factors emphasized by current models of trust judgments. More importantly, subjective consistency is not reducible to a match between the trust ratings of individual elements (objective trust consistency). It is, however, related to a match in the valence of individual components. In fact, subjective consistency mediates the impact of objective consistency in valence on trust judgments. As such, the study shows the importance of considering subjective consistency in predicting trust, and highlights its potential origin in evaluative conflict between individual elements.

## Experiment 2

Experiment 1 documented the role of subjective consistency between two verbal elements of a description of a single target. Experiment 2 examined this issue in a different domain—assessing the competence of neutral targets from pictorial input that varies on consistency. If a similar effect holds in both verbal and visual domains, it would strengthen our confidence in its generality. More importantly, Experiment 2 also tested whether consistency influences trust even if it comes from incidental, background sources that are not intrinsically related to the target (unlike E1). The role of such incidental, background consistency has been suggested in research on cognitive and social judgments but never in the domain of trust^41,56^. In addition, this experiment assessed the sense of subjective consistency using a more complex scale. Finally, we tested whether the effects of consistency extend to more behavioral measures of trust, such as investment decisions in the classic Trust Game^57^. Again, we ran 3 independent sub-studies (separate participant samples), which independently measured Trust Game behavior (2A), Judgment of Trust (2B), and Subjective Consistency (2C).

### Participants

All participants were Polish and were tested in Polish language. Study 2A had 1088 participants (503 males, MAge = 43.54, SDAge = 15.58). Study 2B had 1069 participants (501 males, MAge = 43.51, SDAge = 15.53). Study 2C had 1111 participants (517 males, MAge = 43.22, SDAge = 15.4). Each participant was only in one of the studies. For all studies, we collected responses by using the Polish Nationwide Online Public Opinion Poll Ariadna. All participants gave informed consent, were informed about the study procedures, and could withdraw from it at any point. Participants responded to our research questions that were embedded in an unrelated larger public opinion survey. In line with Ariadna Poll policy, participants were rewarded with credits that could be exchanged for small gifts. The procedure was approved by the ethics committee of the Robert Zajonc Institute for Social Studies at the University of Warsaw. All procedures were performed in accordance with the relevant guidelines and regulations.

### Design and procedure

The experiment consisted of three independent computer-based studies, which followed the same design and differed only on the dependent variable. First, we informed participants that their task would be to evaluate either the target presented in the photo (Study 2A on trust behavior, and 2B on trust judgment) or the whole picture (Study 2C on the feeling of subjective consistency). Each participant was presented with a set of 9 slides showing a person or group of people either in their typical or unusual context. In Study 2A and Study 2B participants evaluated visual targets in terms of trust. In 2A trust was operationalized as a participants’ decision on one round of the classic Trust Game^57^. In our version of this game, participants were asked about transferring some, all or none of their hypothetical endowment of 10 Polish Zlotych (PLN, about $3 USD) to a target person shown on the photo, which would then be tripled and the target person would then decide how much to send back. The specific question asked for each photo was “how much money would you transfer to this “person?”, with responses expressed on 1–100 scale (with 100 meaning the entire endowment of 10 PLN). In Study 2B, participants rated trust for each target person on a photo on a 7 points Likert scale (7 = definitely trust). In Study 2C, the same stimulus material was evaluated in terms of subjective consistency, using a 4-item scale. The scale asked about the extent the whole picture is (i) consistent, (ii) understandable, (iii) has something that is off (reverse coded), (iv) logical. The scale was internally reliable with Cronbach’s alpha 0.96 (with the average correlation in-between items Mr = 0.85).

### Stimulus material

The stimuli were 36 specially designed pictures, which consisted either of a single target person or a group of people on different backgrounds. The pictures were collected from open-source repositories on the web. Specific pictures we used are available on OSF website: https://osf.io/mehg5/?view_only=c8ff816466674556a7ff0e4741d658b8

The target of judgment was always clearly indicated with an arrow superimposed on the picture. We varied consistency by including pictures with a wide range of typicality of the surrounding context, as done in scene perception research^58^. Examples include a businessperson on an office background versus a businessperson on a beach background, firefighters on a building background versus firefighters near a waterfall background, nurse on a hospital background versus nurse on a desert background. This allowed us to have a range of target professions, a variety of settings, and a range of levels in consistency.

### Analysis strategy

We analyzed the data on the level of a single picture. That is, for each picture we had its trust measure operationalized in two different manners: trust behavior (from Study 2A trust was operationalized as a decision on a 100-point scale in a single round of the Trust Game) and trust judgement (from Study 2B as an answer on a 7-point Likert scale). We also had each picture’s subjective consistency rating (from Study 2C). With this information, we mean-centered each variable at the level of a participant—such for every person the mean was 0 and the standard was deviation 1. Hence, we transformed the raw variable from Study 2A to the mean-centered behavioral trust measures; the variable from Study 2B to the mean-centered trust judgement, and the variable from Study 2C to the mean-centered rating of subjective consistency. With the variables transformed to mean-centered measures, we computed the average rating of behavioral trust (from Study 2A), the average rating of trust judgement (from Study 2B), and the average rating of consistency (from Study 2C). Therefore, we had each picture described by the average measure of (i) behavioral trust, (ii) trust judgement and (iii) subjective consistency.

### Results

**Figure 4:**
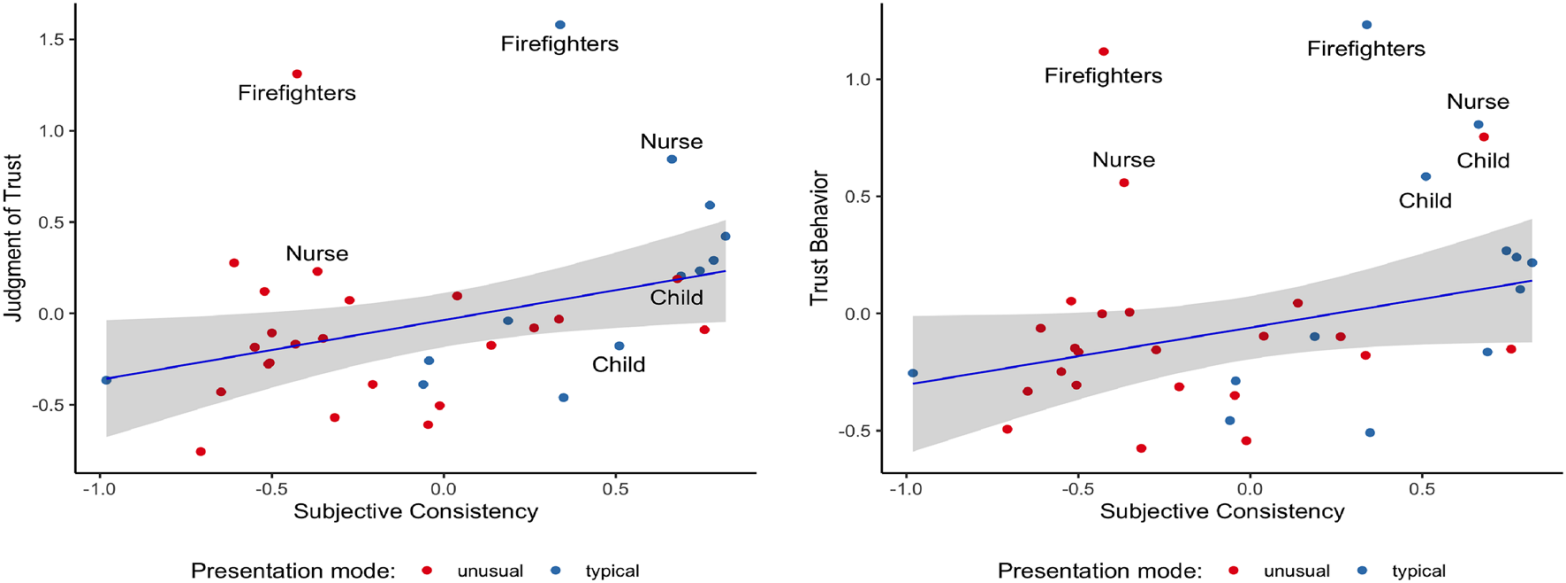
Left Panel: The trend curve with 95% confidence interval for the relationship between Subjective Consistency and Trust Behavior (measured as a decision in a single-round trust game). The GLS model was marginal but the OLS model was significant and Subjective Consistency explained 9.48% of the Trust Behavior variance Right Panel: The trend curve with 95% confidence interval for the relationship between Subjective Consistency and Judgement of Trust (measured as a rating). The Subjective Consistency explained 10.93% of the Trust Judgement variance.

As shown in Fig. 4, subjective consistency was positively related to trust as measured by economic decision and as measured by ratings. In both cases, subjective consistency explained around 10% of variance in trust measures. The figures also make clear that some targets (firefighters, nurses, children) are evaluated particularly high on trust. For statistical assessment, we first examined the relationship between Subjective Consistency and Trust Behavior (both at the level of pictures). The linear model fitted with Ordinary Least Squares explained about 9% of the behavioral trust variance and yielded a significant effect of Subjective Consistency on the Trust Behavior, *F*(1,34) = 4.666, *p* = 0.038. With a 1 point increase of the Subjective Consistency there was a 0.296, [CI 0.0176 0.576]) increase of Trust Behavior, *F*(1,34) = 4.666, *p* = 0.038. A more parsimonious weighted least square linear regression model (weights were inversely proportional to the variance at the level of each picture for Trust Behavior) explained less variance, about 6%. It showed that with a 1 point increase of Subjective Consistency there was a 0.24, [− 0.024, 0.512]) increase of Trust Behavior, *F*(1, 34) = 3.416, *p* = 0.073. It is worth noting here that the OLS model explained more variance (9% vs 6%) than the GLS model. However, the GLS model was more parsimonious than the OLS model. When compared, the former yielded Akaike’s Information Criterion: AIC(df = 3) = 40.702 and the latter yielded AIC(df = 3) = 44.534. Therefore, we presented results for both models for this particular analysis. For all other analyses, the GLS model is superior on both criteria.

Second, we examined the relationship between Judgement of Trust and Subjective Consistency. The model yielded a significant effect of Subjective Consistency on the Judgement of Trust. With a 1 point increase of the Subjective Consistency there was a 0.35 [CI 0.082, 0.628] increase of Judgment of Trust, *F*(1, 34) = 6.96, *p* = 0.012. Both the Ordinary least squares and the Weighted least square models yielded similar results.

## General discussion

This research examined the role of subjective consistency in judgments of trustworthiness based on minimal cues. Those cues were brief verbal descriptions—two words characterizing a single target (Experiment 1) and simple visual information—pictures of targets on different backgrounds (Experiment 2). In both experiments, subjective consistency, measured with a single item (Experiment 1) or with multiple items (Experiment 2), positively predicted judgments of trust (both experiments) and trust behavior (E2). Both experiments showed that subjective consistency is a unique predictor of trust.

Importantly, Experiment 1, which manipulated descriptive and evaluative consistency, shows that a unit of increase in subjective consistency is worth a unit of increase in objective valence and objective trust. This matters because knowing the subjective consistency of the target description predicts as well as knowing the mean valence ratings and mean trust ratings for individual components—factors so far emphasized by current psychological models of trust judgments. Furthermore, subjective consistency cannot be reduced to a simple match (uniformity) between individual elements, either on the dimension of trust or the dimension of valence. This fits with the classic insights from psychology that in impression formation the whole is not a simple sum of the contributing parts, but functions more like a "gestalt"^35^. Our results also fit with the classic observation that psychological consistency is not easily reducible to objective consistency^36^. In essence, whether two elements are subjectively consistent appears to depend on their mutual psychological implications, their mutual expectancy, their perceived fit, rather than on whether those individual elements fall on the same side of trust, or carry the same sign on valence (although subjective consistency is related to objective consistency in valence).

The important role of subjective consistency has parallels in social cognition research with work on objective levels of task difficulty vs. subjective feelings of task difficulty, with research emphasizing that understanding subjective feelings is key for predicting judgments [e.g.,^59^,^60^]. We hasten to acknowledge that the relatively minor role of objective content (on both descriptive and valence dimension) in the current research does not imply that content matters little in other contexts. After all, our materials were designed to minimize the role of substantive content that is highly indicative of trust (e.g., no words or pictures described criminals or saints).

Because we found parallel effects on trust judgments and trust behavior (decision in the *Trust Game*), we propose that subjective consistency can serve as input into different outcomes based on first impressions. This interpretation follows a large body of research on subjective experiences and how they can serve as input into a variety of judgments, decisions, and behaviors^61^. More specifically, we suggest that cognitively, the feeling of consistency is associated with a sense of predictability (more on that shortly). Affectively, the feeling of consistency usually feels positive^44^, perhaps because it is associated with fluency—processing ease^62^. In combination, this presumably leads to a more positive initial impression of the target and supports more favorable trust judgments and more trusting behavior. Of course, these assumptions need to be directly tested but they are in agreement with models that highlight both affective and cognitive components of initial trust impressions from minimal facial cues, and the downstream effects of those impressions (rudimentary attitudes) on consequential behaviors^21^.

There are limitations of our studies. It is worth reemphasizing that we designed our studies to minimize the potential role of substantive trust-relevant content (e.g. adjectives like *corrupt, honest, reliable, conniving* or visual cues to that effect, like a picture of a criminal, or a salesperson in front of a shady used car dealership). That is, we cannot make any claims about the relative impact of the implicit sense of subjective consistency versus the impact of explicitly trust-relevant content. Still, we again suggest that subjective consistency may be important when little substantive information is available^16,61^. A related issue concerns the minimalistic nature of information underlying our trustworthiness judgments and the hypothetical nature of our behavioral measure. Because of this, we cannot confidently speculate about the real-world implications of our results, and also about where the rudimentary attitudes of our participants are located on the broad continuum ranging from genuine distrust, via untrust, to genuine trust^15^. In fact, it seems reasonable to assume that it takes more than minimalistic information to develop levels of trust (or distrust) resulting in enduring attitudes and consequential behaviors. Nevertheless, these limitations are common to much research in the tradition of thin-slicing and first impressions based on minimal cues^20,21^.

Note also a methodological limitation stemming from the fact that in both experiments we varied the levels of consistency, but had no truly "objectively" neutral point. Future studies should address whether consistency increases trust or inconsistency decreases trust. It is also worth studying the interplay of consistency with specific content. Again, to return to an earlier example, in comparison to a consistent pair "*a dishonest thief,*" "*an honest thief*" is inconsistent (evaluatively and descriptively), yet more trustworthy. Follow-up research should also directly manipulate the presumed feeling of subjective consistency, or its (mis)attribution to alternative sources, to assess its causal role in an experimental way, without relying on mediational analyses. If subjective consistency is indeed a cognitive or affective "feeling", it should follow general principles governing subjective experiences^61^.

More challenging questions concern the potential role of culture in our findings. That is, we assume that subjective consistency is evoked by universal processes that reflect the general property of the human mind to form expectations and predictions^33^. However, it is also clear that many specific stimulus combinations that lead to inconsistency are culturally dependent (e.g., many French cooks may be slim, and some California businesspersons work on the beach). So, our particular stimulus combinations may be locally bound, since they depend on actual correlations in a particular environment, and also on cultural stereotypes. More fundamentally, there is some evidence of cultural differences in tolerance for certain types of inconsistency^63^. Relatedly, there is also work on individual differences in preference for consistency^64^ and work showing that negative reactions to inconsistency depend on the need for epistemic certainty^65^. In short, cultural and individual differences are exciting topics for future research.

Our research, relying on rudimentary person stimuli, could fruitfully extend to richer sources of consistency and inconsistency (e.g., narratives, multimodal stimuli). It could also move beyond human–human interactions to examine the role of incidental consistency in trust in technology, including robots and AI^6,7^. This is exciting as trust impressions in technology comprise both cognitive and affective components and can stem from minimal cues eliciting a sense of predictability, familiarity, coherence or weirdness^13,66,67^. This focus on minimalistic cues leading to the feelings of consistency could potentially supplement work that focuses on more explicit, cognitive evaluations of dimensions such as competence, reliability, benevolence, and predictability, which derive from more systematic observations^8^. For example, implicit cues to consistency (e.g., unusual device appearance) could be combined with explicit information on device reliability, in the sense of performing consistently and accurately.

Overall, our research suggests that impressions of a simple target (e.g., a slim cook; a shy salesperson; or a businessperson on a beach) at least partially reflect a subjective sense of fit between individual features. Theoretically, some factors contributing to the sense of consistency may reflect predictability [slim cooks are surprising,^34^,^47^], causal reasoning [slim cooks may not like their own food,^37^], prototypicality [slim cooks are atypical,^45^], as well as greater processing effort (low fluency) for unexpected combinations^49,68^. Notably, subjective inconsistency may even derive from incidental sources, like the background features explored in Experiment 2. All of them probably contribute to a sense that a perceiver cannot easily form a mental model of the target and thus cannot predict the target’s behavior—something that is essential to the basic meaning of trust^8,69^. In short, the current research suggests that psychological models of trust impressions should incorporate the notion of the social mind as concerned with making implicit as well as explicit predictions about the surrounding world^33,34^.

## Supplementary Information

Supplementary Information.

## Data Availability

Data and materials are available on Open Science Framework under the following link: https://osf.io/p8bym/?view_only=c8ff816466674556a7ff0e4741d658b8".
