# Supplementary material for: Subjective consistency increases trust
Source: Sci Rep. 2023 Apr 6;13:5657. doi: 10.1038/s41598-023-32034-4 (PMC10079673; doi:10.1038/s41598-023-32034-4)
Supplement: Supplementary file 1 — Supplementary Information. [file 41598_2023_32034_MOESM1_ESM.docx]

**Supplementary Materials for**

**“Subjective consistency increases trust”**

**Data availability statement**

All datasets, key materials, and accompanying code are in the Open Science Framework repository: <https://osf.io/p8bym/?view_only=c8ff816466674556a7ff0e4741d658b8>

**Information about how sample size was determined for each study and a discussion of statistical power**

As described in the methods for each experiment, our primary analyses consist of bivariate correlations done on a level of an item (adjectives, nouns, pictures). Different ratings on different dimensions of the item were collected from separate samples of participants (all N > 50 in Experiment 1, and all N > 1000 in Experiment 2). As such, we believe that the most conservative calculation of statistical power consists of a simple correlation coefficient between a rating of an item on subjective consistency and rating of an item on trust. In Experiment 1, we calculate this correlation for 64 items, and in Experiment 2, we calculate this correlation for 36 items. Assuming a large effect size (.5), the required N to achieve .8 power is 23. This calculation was done using G*Power 3.1 software with settings for Exact Test: Bivarariate Correlation (Faul, Erdfelder, Lang, & Buchner, 2007). In short, in both Experiment 1 and Experiment 2 we had sufficient power to detect whether subjective consistency predicts trust ratings.

**Data exclusions, variable selection, participant population, and data quality controls.**

The only data exclusions were during a pilot study, which was done in order to select materials for Experiment 1 and are described below.

We report all variables collected for the purpose of the current research and all conditions. Note that for both Experiments 1 and 2, the measures for current research were included in a large public opinion survey with other items unrelated to the current Experiments (see main Text).

All participants were Polish and were native Polish speakers. All materials were presented in Polish.

We used the professional Public Opinion Panel Ariadna to collect data. The panel uses multiple methods to control quality of participants and their engagement. More information about the panel is available here: <https://www.panelariadna.com/>

**Stimulus material for Experiment 1**

We used assessment by 3 competent judges (community members) to prepare an initial list of consistent versus inconsistent adjective + noun pairs. The competent judges were university students and native Polish speakers who resemble the population of our participants and are fully able to understand all terms used in the stimuli, which were presented in Polish.

For the first pilot study, we chose a sample of 30 popular contradictory adjectives, such as “tall” – “short”, “fat” – “slim”, “fast” – “slow”, “big” – “small”, etc. and asked 88 independent raters to assign each of the adjectives a noun that fits well with it (are consistent). In the instructions, we emphasized that the nouns should relate to humans only. Each of the judges provided 30 adjective – nouns pairings.

From this sample, for each adjective, we arbitrarily chose a set of 6 most fitting nouns – candidates for the final set of consistent adjective-noun pairs. In the second pilot study, 65 independent raters assessed how well (on the scale from 1 to 5) the 30 adjectives fit with each of the 6 nouns chosen in the first pilot study.

The pairs assessed as most descriptively consistent were used in the last study, where for each consistent pair, we created a non-fitting, inconsistent pair, composed of the noun from the fitting pair and a contradicting adjective. This set was finally assessed by 36 competent judges, whose assessments were used as the most consistent-inconsistent adjective-noun pairings.

For the final set, we ended up with 64 pairs, or 16 quadruplets composed of two contradicting adjectives, and four fitting or non-fitting nouns. An exemplary fitting procedure for the adjectives “tall” vs. “short” is depicted in the Table 1. The criterion for the inclusion of both the consistent and inconsistent pairing was >2 difference in their consistency ratings.

**Table 1.** The summary of the stimuli selection procedure.

| **Pilot 1** | **Pilot 2** | **Pilot 3** | **Result** |
| --- | --- | --- | --- |
| **Tall**  basketball player *– 4.48*  volleyball player – 4.2  lad – 3.82  sportsman – 3.48  model – 4.08  beanpole – 4.03    **Short**  leprechaun – 3.68  child – 3.23  midget – 3.97  lilliputian – 3.78  pygmy – 3.4  dwarf – 4.06 | **The most consistent pairings:**  tall basketball player *4.48*  short dwarf *4.06* | **Mean assessment of fitting of words in a pairing:**  tall basketball player – 5.64  short basketball player – 2.47  short dwarf – 5.39  tall dwarf – 2.31 | **Final sample based on differences between consistent– inconsistent pairings:**  short dwarf  –  tall dwarf: 3.08 (>2)  tall basketball player – short basketball player: 3.17  (>2) |

**Average inter-item correlations**

For all items from Experiment 1, we first computed correlations between all 64 items (adjective and noun pairings). Therefore, for each item, we had 63 correlation coefficients (the CSV file with the 64 by 64 matrix is available in the OSF repository). Second, we computed the average inter-item correlation as a mean of the correlation coefficients with other items for a given item (see Table 2). The original study was conducted in Polish therefore the items were displayed in Polish. Below we present their translations into English. Because in Polish all nouns are gendered we added information to the translation whether the item referred to a man or a woman. The average inter-item correlation for trust varies between 0.122 for strong male elderly and 0.325 for a male student in a lumberjack shirt with Mean = 0.243 and SD = 0.045. The average inter-item correlation for consistency varies between 0.058 for a short dwarf and 0.371 for a short male basketball player with Mean = 0.261 and SD = 0.08.

**Table 2.** The average inter-item correlations for a given item (adjective and noun pairing).

| **Item in English** | **Trust** | **Consistency** |
| --- | --- | --- |
| a male athlete eating in fast-food restaurants | 0.194 | 0.138 |
| a male athlete caring about his fitness | 0.241 | 0.327 |
| a male driver eating in fast food restaurants | 0.274 | 0.339 |
| a male driver caring about his fitness | 0.3 | 0.207 |
| an obese female model | 0.17 | 0.139 |
| a fit female model | 0.193 | 0.361 |
| an obese female cook | 0.254 | 0.3 |
| a fit female cook | 0.205 | 0.26 |
| a female singer attending receptions | 0.258 | 0.367 |
| a female singer smoking cigarettes | 0.25 | 0.301 |
| a female writer attending receptions | 0.298 | 0.349 |
| a female writer smoking cigarettes | 0.278 | 0.31 |
| a sweat-smelling male model | 0.202 | 0.289 |
| a perfume-smelling male model | 0.249 | 0.168 |
| a sweat-smelling male worker | 0.307 | 0.338 |
| a perfume-smelling male worker | 0.279 | 0.325 |
| a male soldier in a lumberjack shirt | 0.224 | 0.16 |
| a male soldier in an army uniform | 0.236 | 0.22 |
| a male student in a lumberjack shirt | 0.325 | 0.369 |
| a male student in an army uniform | 0.301 | 0.292 |
| a cleaning lady wearing jewelry | 0.234 | 0.241 |
| a cleaning lady wearing slippers | 0.232 | 0.234 |
| a female manicurist wearing jewelry | 0.288 | 0.266 |
| a female manicurist wearing slippers | 0.293 | 0.268 |
| a woman smoking a pipe | 0.21 | 0.212 |
| a man smoking a pipe | 0.289 | 0.239 |
| a woman with bleached hair | 0.179 | 0.273 |
| a man with bleached hair | 0.256 | 0.308 |
| a male conductor wearing sneakers | 0.283 | 0.224 |
| a male conductor wearing oxford's | 0.309 | 0.198 |
| a male rock star wearing sneakers | 0.303 | 0.324 |
| a male rock star wearing oxford's | 0.238 | 0.303 |
| a female pale tourist | 0.263 | 0.19 |
| a female tanned tourist | 0.241 | 0.244 |
| a pale female anemic | 0.169 | 0.335 |
| a tanned female anemic | 0.143 | 0.333 |
| a self-confident male virgin | 0.231 | 0.191 |
| a shy male virgin | 0.26 | 0.173 |
| a self-confident salesman | 0.236 | 0.342 |
| a shy salesman | 0.186 | 0.322 |
| a tall male dwarf | 0.191 | 0.156 |
| a short male dwarf | 0.267 | 0.058 |
| a tall male basketball player | 0.298 | 0.361 |
| a short male basketball player | 0.232 | 0.371 |
| a slow male elderly | 0.23 | 0.12 |
| a fast male elderly | 0.275 | 0.307 |
| a fast male sprinter | 0.224 | 0.225 |
| a slow male sprinter | 0.313 | 0.067 |
| a humble male servant | 0.241 | 0.339 |
| a self-confident male servant | 0.311 | 0.352 |
| a humble security man | 0.229 | 0.192 |
| a self-confident security man | 0.24 | 0.184 |
| a strong male elderly | 0.122 | 0.311 |
| a weak male elderly | 0.206 | 0.273 |
| a strong man | 0.205 | 0.35 |
| a weak man | 0.183 | 0.236 |
| a talkative male solitary | 0.297 | 0.289 |
| a taciturn male solitary | 0.235 | 0.125 |
| a talkative male celebrity | 0.254 | 0.29 |
| a taciturn male celebrity | 0.212 | 0.344 |
| a chubby male glutton | 0.205 | 0.228 |
| a fit male glutton | 0.206 | 0.331 |
| a chubby female model | 0.23 | 0.136 |

**Stimulus Material for Experiment 2**

Pictures used in Experiment 2 are available in the OSF repository: <https://osf.io/p8bym/?view_only=c8ff816466674556a7ff0e4741d658b8>
